# Open access: the view of a commercial publisher

**DOI:** 10.1111/j.1538-7836.2006.02009.x

**Published:** 2006-07-01

**Authors:** A ROBINSON

**Affiliations:** Blackwell Publishing Oxford, UK

## Abstract

*See also* Barbour V, Patterson M. Open access: the view of the Public Library of Science. This issue, pp 1450–3.

Believers in open access (OA) argue that the subscription-based journal model is like a clot blocking the free-flow of scientific research to vital research organs and the public, cutting off the supply of ideas and innovations.

But believers in traditional journals argue that, with a single cut, there is a real risk that scientific research will leak in an uncontrolled fashion that would be impossible to stem. The end result will be an undifferentiated pool of unreviewed research, which will, because of its lack of structure, not only halt the diffusion of innovation to the same vital research organs, but also challenge the viability of the whole body by affecting other systems, such as peer review and societies like the International Society on Thrombosis and Haemostasis.

I will argue for a delicately balanced system that allows research that is published in journals to flow to the organs that need it, rapidly and efficiently. But I will also argue for a process of evolution, not revolution, in a spirit of experimentation, to safeguard what works well now, but also to ensure that neither sustainability nor quality is compromised.

## What is OA?

Open access is when ‘the author grants to all users a free, irrevocable, worldwide perpetual right of access to, and a license to copy, use distribute, transcript, and display the work publicly in any digital medium for any responsible purpose, subject to proper attribution of authorship’.

From a purist's perspective, it generally manifests itself in two ways.

1. ‘Author-pays’ journals that allow anyone to access all articles for free. They do not levy subscription charges, but cover the cost of publication through subsidies, sponsorship, or by charging authors, the authors’ funding body or employer, a fee for publication.
2. Institutional or subject repositories, which are online collections of materials, including research papers. They are often managed by a university, institution, or funding body, and tend not to offer peer review (leaving publishers to carry the cost of organizing the review process).

Many ‘traditional’ publishers of journals, including societies, have also incorporated aspects of the OA debate into their own publishing, creating an array of hybrid experimental models.

So where did OA come from? There are five strands to the OA argument:

1. the library-funding crisis;
2. that lack of access impedes research;
3. the right to access publicly funded research;
4. the needs of the developing world; and
5. the profits of scholarly societies and publishers.

## The library-funding crisis

Librarians have been active proponents of OA because they see it as a solution to their funding crisis. They believe there are too many journals (around 20 000 peer-reviewed titles) publishing too many papers, and they are too expensive: so no library can possibly afford to stock them all.

This volume of research comes from a research community that has been funded well in excess of inflation. Journal price increases correlate with increases in research funding, which feed through to an increase in the number of articles published. The European Union is targeting an increase to 3% of Gross Domestic Product (GDP) for research and development (R&D) spend, and Asia is following fast, with Pakistan increasing the number of PhD students from 250 to 1000 graduating per year. Peer-reviewed article output could easily rise from 1.5 to 2.5 million articles in the next 5 years. Universities must decide whether to invest in research (generating grant income) or the dissemination of research. Library funding has been the casualty, with budgets falling from 4% of university expenditure to just 2%.

Librarians and publishers have reacted by buying and selling e-journals in bulk: libraries club together to form consortia that buy e-journal collections from the large publishers. The ‘Big Deal’ (a collection of titles bought at high discount on top of the existing locally held subscriptions that are bought direct) has pros and cons, but it has exploded online access to journals at incremental cost. In the USA, between 1998 and 2003, R&D spend increased by an average of 9.15% per annum, while the Association of Research Libraries’ spending increased by an average of 7.16%, the unit price of journals increased by an average of just under 1%, and the number of titles subscribed to increased by an average of 5% (estimates from Jan Velterop of Springer, pers. obs on a library listserve).

Many publishers also license journals to aggregators, such as OVID and EBSCO, who then sell databases of content to libraries, and grant access to libraries in poorer countries through initiatives such as HINARI (see below). If we take a mature title, such as *British Journal of Haematology*, as an example, this translates into a circulation of around 10 000 libraries in academic research institutes, hospitals, and colleges. I would argue that there are very few institutions supporting research in hematology that do not have access to it, and thousands more with only a peripheral interest can now gain access where it was impossible only 5 years ago.

Journal prices are just one part of this equation. Librarians also have to tackle their other costs: for every $1 spent acquiring journals, another $2 is spent on overheads. Even if all journals became free tomorrow, the library-funding crisis would hit again within 10 years [1]. Meanwhile, libraries are no longer the ‘must-go’ destination for researchers, who ranked libraries 11 of 12 routes for discovering research [2]. If OA publishing is one route to balancing the budgets of libraries, certainly there are other options just as close to hand.

## Lack of access impedes research

Online availability of journals has heightened researchers’ appetite for still more content. There are now a multitude of routes to access content and mind-blowing tools for uncovering research that would otherwise have gone unnoticed. It is unacceptable to some that access is denied under any terms [3].

In reality, researchers have never had it so good. A recent survey of North American and European microbiologists and immunologists showed that 90% agreed that convenient access to journal full-text online had enabled them to become more effective researchers (I. Rowlands and R. Olivieri, pers. obs.). In addition, 97% agreed that digital library platforms had saved them considerable time in finding and retrieving articles.

Unlimited access will not provide a linear increase in research awareness and productivity – there is a law of diminishing return. When researchers were asked to rank the factors that would promote, rather than inhibit research productivity, access to a wider range of e-journals was ranked 12 of 16 factors (I. Rowlands and R. Olivieri, pers. obs.). More funding, the ability to recruit suitable research assistants, initial ‘seed corn’ funding, more autonomy in research direction, and cutting red tape were cited as the top five promoters of research productivity.

Open access advocates also argue that limiting access to research inhibits readership, thus reducing citations and research impact (and therefore limiting career prospects). This is not the case, according to Thomson-ISI, the creators of the journal impact factor. The ISI database contains nearly 200 OA journals, of which they analyzed 148 journals in the natural sciences, concluding that ‘To date, no clear effect has been observed. Though there is some suggestion in aggregate of a slightly more rapid accumulation of citations, this effect is, so far, minimal. The wide distribution of these OA journals has not yet been shown to have any appreciable effect on their appearance in lists of cited references in other journals’ [4].

## Public access to publicly funded research

The public should have access to publicly funded research data published in biomedical journals, especially when it is a patient or relative seeking information about the latest treatments.

Why? The scholarly communication system is not designed for communication between researchers and the public. Better channels already exist to do that, including television, radio, newspapers, patient information from medical societies and charities, such as the World Federation of Hemophilia, a plethora of health websites, and last, but not least, actually talking to your doctor. To subvert a system created to enable peer-to-peer communication is not doing patients, or their relatives, any favors, especially when 90 million people in the USA have trouble understanding and acting on health information [5]. A healthy supplement to the existing system is the patientINFORM project, in which the American Heart Association (owners of *The Journal of Arteriosclerosis, Thrombosis and Vascular Biology* [ATVB]), the American Diabetes Association, and the American Cancer Society create bespoke news pieces relating to important papers published in the hundreds of journals (including this *Journal*) of 23 medical publishers and societies [6].

The second bone of contention is that copyright should not be transferred to the publisher. To misquote Mizner: ‘If you steal from one author, it's plagiarism; if you steal from many, it's publishing’. Surveys show that authors do not attach much importance to retaining copyright in an article [2]. It was ranked 10 of 10 factors when considering where to publish an article. In a survey of just under 2000 authors and readers of this *Journal* (with a 20% response rate) just 36% felt that retaining copyright was either a ‘very important’ or an ‘important’ factor when choosing where to publish.

For the small minority of authors who do care, or whose funder tells them to care, many journals and publishers now have more liberal copyright policies. A large proportion of Blackwell Publishing journals have, therefore, adopted an Exclusive Licence Form (ELF) in place of the old Copyright Assignment Form. With an ELF, the author retains the intellectual copyright but allows the journal to retain the commercial rights.

Taxpayers do have a right to know where their taxes are spent, but strong-arming the entire journal system to conform jeopardizes the considerable amount of research that is not government-funded. Evidence shows that a third of authors (more than 50% in medicine) publish most of their work without external funding [2]. A quote from one survey respondent illustrates the point nicely: ‘Most publishing of medical articles is done by people with no grant money and no institutional support. If the author were forced to pay this would inhibit much of this output’.

How many *Journal of Thrombosis and Haemostasis* (JTH) authors are publicly funded? In the 16 issues from January 2005 to April 2006, 42% of the articles were written by authors who did not cite any funding support. From the JTH survey 39% said their work was either only partially funded or completely unfunded. Of the more than 250 sources of funding for JTH papers, the NIH funded 78 of the 736 items published in the same period. That is 11%, and confirms a very dispersed source of funding dominated by small charities, or researchers cobbling together the funds to support their own work. The prospect of all authors paying for publication simply is not an option.

Authors do not want to (and indeed most cannot afford to) pay for the cost of publication; they think that a greater burden should be borne by research funders, sponsors, and government [2].

Let us take the world's largest funder of biomedical research: the NIH. The cost to the USA taxpayer of PubMed Central (PMC) was $1 million in 2005, and would be $3.5 million if there was full compliance, with every NIH-funded researcher depositing articles as requested [7]. As Ann Okerson, the influential librarian from Yale University, questions: ‘To move towards government support at just the moment when National Institutes of Health and National Science Foundation funding is flattening out and growing more difficult to obtain feels particularly risky’, and ‘to surrender a diverse funding base for a few payers or to ask a small number of research-intensive institutions to support publication for all could actually increase the risk of serious contraction or chaos in the availability of information’ [8]. Surely, there could be no threat to the future of the NLM or PMC? Well, the US Environmental Protection Agency is slated to shut down its network of libraries and its electronic catalogue as a result of budget cuts. But another issue comes into play: the government funds the research, and now controls its dissemination. What happens if the research does not square with the current political agenda? As Okerson [8] points out: ‘Are we already too dependent on government regulation or, as some would say, interference'?

So if a diverse funding base is a good thing for dissemination, take JTH as an example. It has more than 250 separate sources of funding, and more than 60% of these funded just one paper from a wide range of institutions and universities. The cost of implementing an institutional repository in a university on DSpace (open-source software requested by MIT for archiving eprints and other kinds of academic content) is reckoned to be in excess of $50 000. Given that libraries across the globe are now gearing up to launch institutional repositories, it is easy to see how these costs can escalate. If each institution takes responsibility for disseminating its research, this could lead to a very fragmented research base, and as a consequence some research might be lost. Even if Google™ Scholar can retrieve most of it now, who knows what will happen in 10 years’ time, when an institution might decide that it can no longer support the financial burden? We have to be absolutely certain that the systems advocated by OA will survive the vagaries of current political and taxpayer opinion: the full-text of the world's first English language journal, *The Philosophical Transactions of the Royal Society* has endured for 341 years.

## Developing world access

Open access to research literature would significantly improve the quality of health care and scientific research in some of the world's poorest nations, or so the story goes. So, what about HINARI, the Health InterNetwork Access to Research Initiative that is jointly administered with the World Health Organization? This philanthropic initiative was launched in 2002, and makes available online across 1590 institutions in 113 countries, over 3230 journals from more than 60 publishers, including JTH and 210 other Blackwell medical journals [9]. So, the subscription is not the problem, and yet, in reality, usage of JTH is depressingly low. How can this be, if there is an insatiable appetite for knowledge that can only be met by OA? Maybe it is because the basic infrastructure is absent. This quote from one recipient in Ecuador paints a vivid picture: ‘We are a small hospital with approx. 30 people working in it and serving the community. We don't have a library but we have 3 computers with one of them having Internet. Not very fast but useful. Doctors here and nurses would benefit a lot from a service like this since I'm trying to teach them to use the Internet to gain access to the latest medical information’ (March 4, 2004).

Subscription-based publishers are working through HINARI and other similar organizations on helping with training and infrastructure.

## Publishers and societies make profits

At least some of the OA fervor derives from the perception that publishers and societies are making unjustifiable profits from journal publishing. There are thousands of journals published by societies, not-for-profits, and ‘commercial’ companies. Blackwell, for example, is the world's leading publisher for societies and ‘is viewed, by some, as an “honorary not-for-profit publisher”’ [3]. Many societies invest heavily in their niche community by running conferences, educating members (through guidelines, continuing medical education, continuing professional development, travel scholarships), funding public advocacy, providing patient information, and even funding research.

Blackwell Publishing's research shows that receipt of the society's journal is seen as one of the top two benefits of membership, along with the society conference. Without this member benefit and income from the journal, some societies may well close, and it is not clear whether government, universities or healthcare providers will fund these other invaluable services. Some societies will seek other sources of income, which could include significant sponsorships from the pharmaceutical industry. Many question whether this level of financial dependence would be healthy.

Not surprisingly, therefore, societies are asking for caution in the rush to provide OA. Before we throw out the ‘baby with the bathwater’, we need to be quite sure that this new model of scholarly communication will be sustainable.

## The impact of OA

Sustainability is a vital question in this debate, as is the recognition that there is a cost that has to be met by someone. OA supporters have thrown two new models into the publishing mix to try and ensure more equitable dissemination of research: the ‘author-pays’ model and self-archiving. Taken in isolation, neither of these will create a system that is demonstrably superior. Existing journals and publishers, however, have incorporated these two models into their own traditional approaches, creating hybrids that offer potentially viable compromises.

## The author-pays model

The awareness of author-pays OA journals among the author community is rising: across a range of disciplines 30% claim to know ‘quite a lot’ or ‘a lot’ (12% more than a year ago) [2]. Authors also claim to be publishing in more OA journals than before: 29% in 2005, up from 11% in 2004 [10].

So how do these perceptions stack up against reality and what is the uptake in the field of thrombosis and hemostasis?

The Directory of Open Access Journals (DOAJ) contained 2127 author-pays titles on March 20, 2006 [11]. A search in DOAJ identified 216 papers in thrombosis and 26 papers in hemostasis, and two OA journals: *Thrombosis Journal*, and the *Journal of Atherosclerosis and Thrombosis*. In 2005, these two titles published 76 items: this represents 14% of what JTH published in the same period, less than two issues, or just 2% of the 3500 thrombosis and hemostasis papers published in 2005 within journals listed in the ISI database. So the uptake seems remarkably low, perhaps suggesting that authors may think they are publishing in OA journals when in fact they are not.

How do these author-pays journals compare to their more traditional competitors? There has only been one extensive comparison of OA journals, commissioned by the Association of Learned and Professional Society Publishers, HighWire Press, the American Association for the Advancement of Science, with contributions from the Association of American Medical Colleges [12]. In *The Facts about Open Access*, which surveyed almost 500 full OA titles, it transpires that of the journals surveyed:

1. 52% of OA journals do not raise any author-side charges at all, and are far more dependent on other sources of income, such as advertising and sponsorship;
2. over 40% of OA journals are not yet covering their costs: financial viability was often a low priority;
3. the journals had substantially lower rates of article submission (<10% of the non-OA journals), but were less selective, with acceptance rates of over 50% (higher than the non-OA journal cohort);
4. only 72%of articles in OA journals were copy-edited (compared to almost 100% in traditional journals), and OA journals tended to rely heavily on internal editorial staff for peer review (of around 28% of their papers).

These data present a picture of fledgling titles that are struggling to impose themselves on the academic landscape. There is little evidence, so far at least, that the author-pays model is creating a sustainable challenge to the subscription model.

Faced with a hand-to-mouth existence, it is easy to see why ‘traditionalists’ fear that author-pays will ultimately lead to a reduction in standards: if costs cannot be covered by author fees from high-quality papers, then why not publish lower quality articles instead?

The author-pays model discriminates against researchers without grant funding. Ironically, it also discriminates against researchers from low-income countries, who cannot afford the fees. Some OA journals waive these fees, but for how long can that continue in the absence of a viable and sustainable business model?

## Self-archiving of papers in subject or institutional repositories

Many funding agencies now require authors to retain copyright and to make their articles available in a repository, usually within 6–12 months of publication [13–16].

To date, the rate of compliance has been low. Recently, the NIH reported that <4% (1636) of the articles eligible (43 000) for submission in the first 8 months had, in fact, been deposited in PMC. This excludes the 5400 articles published in regular PMC participants, which are deposited automatically. The NIH says‘lack of awareness does not appear to be the primary reason for the low submission rate’ [7]. But the Publishing Research Consortium recently presented data showing that, while 85% of NIH-funded authors have at least heard of the policy, only 18% know ‘a lot’ or ‘quite a lot’ about it [17]. They believe submission rates are low because authors do not know about the process and fail to identify with the benefits, with 20% of authors saying they intended to submit but had not got around to it.

There were 2288 authors’ manuscripts deposited in PMC on April 10, 2006. Of these, six were from JTH, and a handful from *ATVB* (five), *Blood* (11), and *Thrombosis & Haemostasis* (two). Interestingly, two papers from JTH had been published within the last 12 months, and should not have been deposited by the authors (infringing the embargo period specified by JTH). Our survey of JTH authors and readers showed that 16% claimed to have deposited an article in an institutional repository – while a significant minority (27%) said they had no intention of depositing articles in the future.

Awareness of institutional repositories is far lower in JTH authors and readers than it is for author-pays OA: just 13% of respondents claimed to know ‘a little’ or ‘a lot’. This leaves an overwhelming 87% knowing ‘a little’ or ‘nothing at all’. Only 35% of JTH respondents thought repositories would be either ‘very likely’ or ‘quite likely’ to undermine the existing journal system (vs. 57% for author-pays).

This is ironic because ‘traditional’ publishers are far more concerned about the long-term impact of institutional repositories than the author-pays model. Imagine a scenario where approaching 100% of JTH papers are in open archives and available for free. Publishers argue that free online availability of large tracts of research literature is bound to lead to a large-scale move away from libraries paying subscriptions. And it is not just publishers that have made this connection. The Wellcome Trust points out that ‘If any kind of open archive is established, a subscriber-pays system cannot easily survive’. They add that ‘in the medium to long term therefore, that is, the time it will take to establish an effective open archive or series of interlinked, searchable open archives… the question facing journal publishers is not whether to offer OA or not, but how to position their journals so that they are able to continue to play an important part in a world in which OA, through an open archive and very cheap or free document delivery, is the norm’ [18].

## The reaction from existing journals and publishers

So if you think that mass extinction of journals is an overstatement, then think again. Environmental challenge is a great promoter of evolutionary change, so existing journals, publishers, and societies are now working hard to respond in ways that do not completely undermine their sustainability.

*The Facts about Open Access* lists 14 different flavors of publishing model that are being tested out by established journals, mixing subscriptions with full OA and support from author fees, delayed OA, free or charging for archival content, institutional memberships, grants, industry sponsorship, and advertising [12].

A group of mainly North American not-for-profit medical and scientific societies and publishers, known as the DC Principles Group, responded by announcing the Washington, DC Principles for Free Access to Science in 2004 [19]. These include the following commitments:

1. selected important articles are free online from the time of publication;
2. full-text articles should be available within months of publication (may be up to 1 year);
3. research articles should be free to scientists in low-income nations;
4. content should be available for indexing by major search engines so that readers worldwide can easily locate information; and
5. development of long-term preservation solutions.

Most Blackwell journals also impose an embargo period, which in the case of JTH is 12 months, during which authors may not deposit their article in a repository. The relative usage of JTH articles after publication is a curve typical of most biomedical titles, in which there is a spike of usage in the first 12 months, trailing off thereafter. The ISTH, therefore, decided that a 12 month embargo period would protect subscriptions and allow scientists to comply with the requirements imposed by their funders.

In addition, Blackwell Publishing launched Online Open in 2005, which is an equivalent of the author-pays model. For the fee of $2500 the author is assured of full OA to the article immediately after publication. Online Open is an ongoing experiment in which 82 journals are participating (not including JTH). To date 17 papers have been published and a further 47 are in press. To put this into perspective, this represents <0.01% of the total papers published by Blackwell in 2005.

## A world without journals

Publishers and societies are making strenuous efforts to find new ways of optimizing the publication and free-flow of research. But the threat to journals, whether intended or not, is real. If journals were to become extinct tomorrow, how would the academic community have to pick up the pieces? Put another way, the activity of ‘publishing’ in the context of the scholarly communication system has a cost that will continue regardless of the existence of journals or publishers – so what value do publishers and journals add?

In 2005, 3500 papers were published in the area of thrombosis and hemostasis. Disregarding those rejected for publication, and assuming that each accepted paper had an average of two peer reviewers, the research community performed at least 7000 reviews. And they did it quickly in many cases – averaging 28 days to a first decision for JTH. The net result was a research pool that was better presented, sparing the community the trouble of reading some work that was, frankly, not up to scratch.

JTH authors value peer review very highly when considering where to publish, with 96% citing it as a ‘very’ or ‘quite important’ factor. Taking journals out of the equation altogether, will 7000 individual reviews spontaneously happen in a timely and orderly fashion? I think not.

Even then, peer review is only part of the editorial process. A good journal is much more than the sum of its parts. JTH publishes a wide variety of information in a range of formats including debates, reviews, and commentaries, which are actively commissioned and chased up by the editorial team. For many readers these are more important than the original research.

In JTH, 60% of papers come from more than 40 non-English speaking countries. In some cases, journal editors see the germ of quality research that is obscured by unclear English, and work with the author to improve its presentation. In a low-cost database world, this research would be passed over as most readers cannot invest the same effort.

Then there is the typesetting to create the pages that 58% of JTH readers find online but printout to read. Typesetting also introduces a wealth of content-tagging to create electronic files that link to other content, including external databases. And what of printed journals? Most OA advocates assume print will disappear, but it is still wanted by many customers. Several studies have shown that medical faculty still value print above online journals, because they are more mobile and less fixed to a single desk in a laboratory.

Open access databases could perform search and retrieve as well as any journal, but journals are a shorthand for value and an understanding of what is and is not worth reading. Even the Wellcome Trust concedes that: ‘Journals provide a framework through which readers assess the value of articles and it is difficult to conceive of a system without a journal-like institution. A collection of articles without the quality measure given by publication in a journal would be less valuable. Articles could be individually kite-marked but readers would not have the sense of perspective and orientation which a journal gives and, without the journal, search costs for readers would be much higher’ [18].

Researchers attach great value to being able to reach deeply into specialist readerships for their articles [2]. In our JTH survey, 92% of respondents said that journal readership was either a ‘very’ or ‘quite important’ factor when deciding where to publish their papers. For many researchers this is not about mass readership – it matters more that 200 like-minded peers do see their paper, than that the rest of the world could see their paper but ignore it.

Finally, like it or not, journals have an established role in the assessment of research impact and productivity. But it is more than simple metrics: researchers and their employers want their high-quality efforts to rub shoulders with other quality work, with a stamp of approval from accredited experts. Publishers and societies have spent decades building the quality of their journals, establishing their reputations and brands.

Any researcher can publish on the web – but what authors want more than anything else is the peer recognition, which is why 98% of JTH authors value journal reputation above every other factor when choosing where to publish.

## Conclusion

I have argued that journals are not the principal barrier to the free-flow of scientific research. Library budgets need to match investments in R&D. Research funding needs to be better organized, and red tape cut. The IT infrastructure in the developing world needs to be upgraded. The public need patient-friendly information about government-funded research.

The OA debate has focused on two solutions, neither of which creates a viable, sustainable business model proven to be more effective than the current system. Author-pays journals discriminate against unfunded researchers, from the developed or developing world. Very few OA journals are financially viable without donations and sponsorships. Self-archiving could seriously undermine subscription-based journals, risking the loss of decades of quality-controlled expertise in publishing high quality, peer-reviewed content. The funders of research are not necessarily the best guardians of it in the longer term, and research will become fragmented and more time-consuming to sift.

Meanwhile, societies and publishers are energetically engaging in the debate and have begun active experimentation with a range of business models. As the Royal Society says: ‘The worst-case scenario is that funders could force a rapid change in practice, which encourages the introduction of new journals, archives and repositories that cannot be sustained in the long term, but which simultaneously forces the closure of existing peer-reviewed journals that have a long-track record for gradually evolving in response to the needs of the research community over the past 340 years. That would be disastrous for the research community’ [20].
